# Antibodies Specific to Membrane Proteins Are Effective in Complement-Mediated Killing of Mycoplasma bovis

**DOI:** 10.1128/IAI.00740-19

**Published:** 2019-11-18

**Authors:** Yun-ke Zhang, Xia Li, Hao-ran Zhao, Fei Jiang, Zhan-hui Wang, Wen-xue Wu

**Affiliations:** a Key Laboratory of Animal Epidemiology and Zoonosis, College of Veterinary Medicine, China Agricultural University, Beijing, China; b China Animal Disease Control Center, Beijing, China; Washington State University

**Keywords:** *M. bovis*, MI, complement, MAC, bacterial lysis

## Abstract

The metabolic inhibition (MI) test is a classic test for the identification of mycoplasmas, used for measuring the growth-inhibiting antibodies directed against acid-producing mycoplasmas, although their mechanism still remains obscure. To determine the major antigens involved in the immune killing of Mycoplasma bovis, we used a pulldown assay with anti-M. bovis antibodies as bait and identified nine major antigens. Among these antigens, we performed the MI test and determined that the growth of M. bovis could be inhibited effectively in the presence of complement by antibodies against specifically membrane protein P81 or UgpB in the presence of complement. Using a complement killing assay, we demonstrated that M. bovis can be killed directly by complement and that antibody-dependent complement-mediated killing is more effective than that by complement alone. Complement lysis and scanning electron microscopy results revealed M. bovis rupture in the presence of complement. Together, these results suggest that the metabolic inhibition of M. bovis is antibody-dependent complement-mediated killing. This study provides new insights into mycoplasma killing by the complement system and may guide future vaccine development studies for the treatment of mycoplasma infection. Furthermore, our findings also indicate that mycoplasmas may be an appropriate new model for studying the lytic activity of membrane attack complex (MAC).

## Abstract (precis)

The metabolic inhibition (MI) test is a classic test for the identification of mycoplasmas, used for measuring the growth-inhibiting antibodies directed against acid-producing mycoplasmas, although their mechanism still remains obscure. To determine the major antigens involved in the immune killing of Mycoplasma bovis, we used a pulldown assay with anti-M. bovis antibodies as bait and identified nine major antigens.

## INTRODUCTION

Mycoplasmas are a group of bacteria belonging to the class *Mollicutes*; they lack cell walls, have the smallest bacterial genomes, and presumably evolved from Gram-positive bacteria via degenerative evolution (1). Despite their apparent simplicity, over 200 mycoplasma species have been identified to date. Because they can infect humans as well as many economically important animals, they are of great importance in both the medical and veterinary fields (2). In cattle, Mycoplasma bovis leads to a variety of clinical manifestations, including bronchopneumonia, otitis, genital disorders, arthritis, mastitis, and keratoconjunctivitis (3). These pathogens often lead to chronic infections, and the best solution for controlling this disease would be the development of safe and effective vaccines (4).

The metabolic inhibition (MI) test is a classic mycoplasma identification method that was first described by Jensen in 1964; it is similar to virus neutralization and is used for measuring growth-inhibiting antibody (5). The acid production resulting from mycoplasma growth leads to a decrease in the pH and a consequent color change of culture medium containing a pH indicator (6). Previous research has shown that the immune killing of some mycoplasma species requires complement, although the mechanism still remains obscure (7, 8).

The complement system, which consists of multiple proteins, is a crucial part of the innate immune system and emerged more than a billion years ago. It plays important roles in defending against pathogen infections and regulating adaptive immunity (9, 10). Bacteria can trigger all known routes of complement activation (i.e., the classical, lectin, and alternative pathways) through the recognition of conserved bacterial structures, resulting in the formation of the membrane attack complex (MAC), which has a split-washer shape comprising different complement components (C5b, C6, C7, C8, and multiple copies of C9) (11–13). Generally, the MAC is thought to kill Gram-negative bacteria by direct lysis or by destroying the metabolic system (14–17). However, the ability of the MAC to directly kill bacteria remains under debate. The critical question is how the MAC disturbs both the inner and outer membranes. Additionally, the MAC is presumably ineffective at the direct killing of Gram-positive bacteria, due to the thick peptidoglycan (PG) layer (18). Furthermore, the sublytic complement components C5b to C9, apart from their classical role of lysing cells, can also trigger a range of nonlethal effects on cells, generally inducing inflammation (19–21).

This report describes the production of a rabbit polyclonal antibody (pAb) that inhibits the growth of M. bovis *in vitro* and the identification of major antigens recognized by the pAb, determined using a pulldown assay with anti-M. bovis antibodies as bait. Next, we prepared rabbit antiserum against the PDHE2, purine nucleoside phosphorylase (PNP), P81, and UgpB proteins of M. bovis, which were expressed by an Escherichia coli expression system. The protective proteins screened by MI are candidate antigens constituting a major research effort toward the development of an effective vaccine. Furthermore, we found that M. bovis can be killed directly by the complement system. These results indicate that mycoplasmas may be an appropriate new model for studying the mechanism of the lytic activity of the MAC.

## RESULTS

### Screening and identification of dominant antigens.

**FIG 1:**
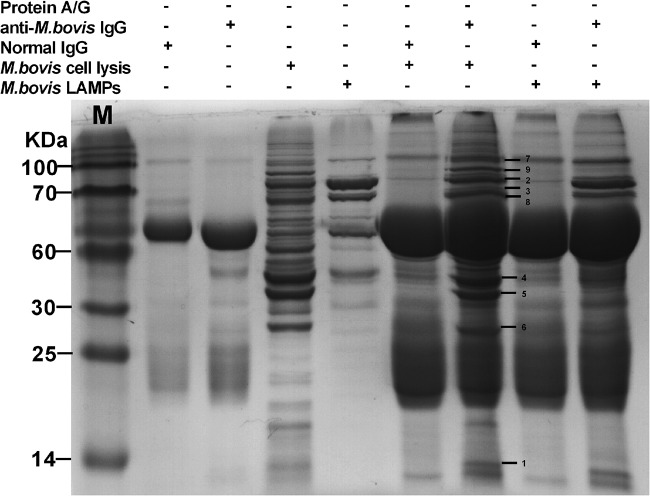
Pulldown of major antigens of M. bovis. To identify the major antigens of M. bovis, a pulldown assay was performed using rabbit anti-M. bovis antibodies or normal rabbit antibodies (negative control) on extracts of M. bovis or M. bovis LAMPs. The resulting pellets were examined by SDS-PAGE.

Hyperimmune serum derived from rabbits immunized with whole M. bovis was screened by indirect enzyme-linked immunosorbent assay (iELISA). This study aimed to determine the major antigens of M. bovis responsible for the induction of specific antibodies that can inhibit mycoplasma growth using the MI test. We hypothesized that the major antigens of M. bovis could be pulled down by specific antibodies in rabbit anti-M. bovis serum. To test this, we performed a pulldown assay using rabbit anti-M. bovis antibodies or rabbit normal antibodies on bacterial cell extracts of M. bovis and lipid-associated membrane proteins (LAMPs). On the resulting gel, at least nine protein bands were visible clearly for lanes that contained M. bovis lysate or LAMPs incubated with rabbit anti-M. bovis antibodies compared with the lanes that contained samples incubated with rabbit normal antibodies (Fig. 1). Analysis by liquid chromatography-tandem mass spectrometry (LC-MS/MS) of the amino acid sequences of these bands identified nine proteins (Table 1).

**TABLE 1:** Identification of the target proteins by LC-MS/MS^*a*^

Spot no.	Protein name	Protein abbreviation	GenBank accession no.	MW (kDa)	Protein score
1	Pyruvate dehydrogenase E2 component	PDHE2	AIA34105.1	8.59	811
2	Membrane lipoprotein P81	P81	AIA34118.1	81.60	20076
3	Glycerol ABC transporter, glycerol binding protein	UgpB	AIA34272.1	70.12	7213
4	Pyruvate dehydrogenase E1 component subunit alpha	PDHA	AIA33689.1	41.33	13433
5	Pyruvate dehydrogenase E1 component subunit beta	PDHB	AIA33690.1	36.18	13774
6	Purine nucleoside phosphorylase	PNP	AIA34127.1	25.92	6237
7	Phosphoketolase	PK	AIA33718.1	89.94	9651
8	Hypothetical protein		AIA33828.1	69.82	9078
9	Lipoprotein		AIA34109.1	85.52	8954

a The target protein bands were cut out after separation by SDS-PAGE and subjected to liquid chromatography-tandem mass spectrometry (LC-MS/MS). MW, molecular weight.

### Purification and identification of recombinant proteins.

**FIG 2:**
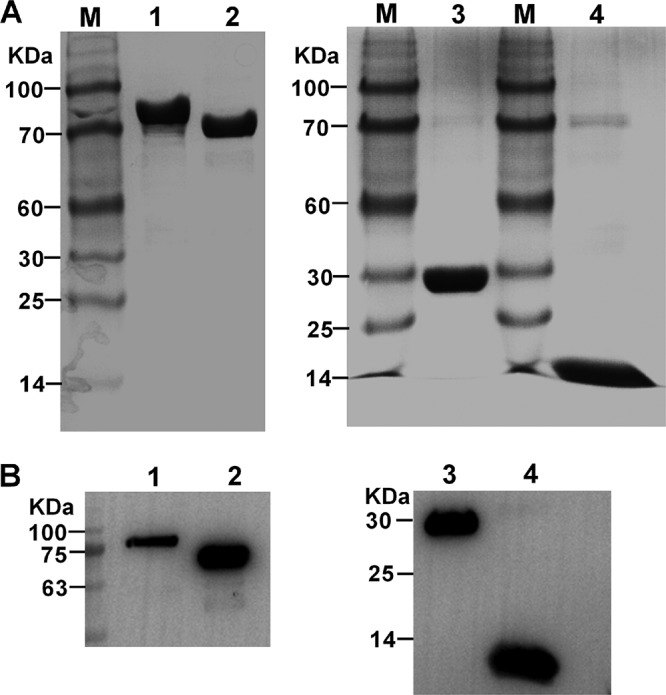
Purification and identification of M. bovis recombinant proteins. Purified recombinant proteins were identified by SDS-PAGE (A) or Western blotting with rabbit anti-M. bovis antibodies (B). Lane M, molecular weight marker; lane 1, purified recombinant P81; lane 2, purified recombinant UgpB; lane 3, purified recombinant PNP; lane 4, purified recombinant PDHE2.

Recombinant P81 (rP81), rUgpB, rPNP, and rPDHE2 were expressed as inclusion bodies and purified by His tag Ni afﬁnity chromatography; SDS-PAGE revealed that they had molecular weights of 80 kDa, 70 kDa, 25 kDa, and 10 kDa, respectively (Fig. 2A). All four purified recombinant proteins could be recognized by rabbit anti-M. bovis antibodies (Fig. 2B). This result indicates that they are all major immunogenic antigens of M. bovis.

### Rabbit antiserum IgG titers.

Antisera with high specificity were produced after rabbits (*n* = 3) were immunized with any of the four purified recombinant proteins. The IgG titers in the various rabbit antisera were detected by indirect ELISA using M. bovis extracts as the coating antigens as well as by agar gel diffusion precipitation and indirect hemagglutination inhibition assay (Table 2). These antiserum strongly reacted with proteins corresponding to size in the M. bovis lysates (Fig. S1). The results of these assays indicate that the immunization of rabbits with rPDHE2, rPNP, rUgpB, or rP81 elicited robust humoral responses and that these antisera are monospecific against these antigens.

**TABLE 2:** IgG titers of the rabbit antiserum^*a*^

Serum	IgG titer by:		
	iELISA	AGP	IHA
Anti-PDHE2	1.0 × 10^5^	8	6,400
Anti-PNP	1.0 × 10^5^	8	3,200
Anti-UgpB	4.0 × 10^5^	8	3,200
Anti-P81	1.6 × 10^6^	8	3,200

a Each purified recombinant protein was separately injected subcutaneously into New Zealand rabbits, serum was harvested after the last injection, and the titer of the serum was determined via indirect enzyme-linked immunosorbent assay (iELISA) using M. bovis extracts as the coating antigens as well as by agar gel diffusion precipitation (AGP) and indirect hemagglutination assay (IHA).

### Subcellular localization of PDHE2, PNP, UgpB, and P81 in M. bovis.

**FIG 3:**
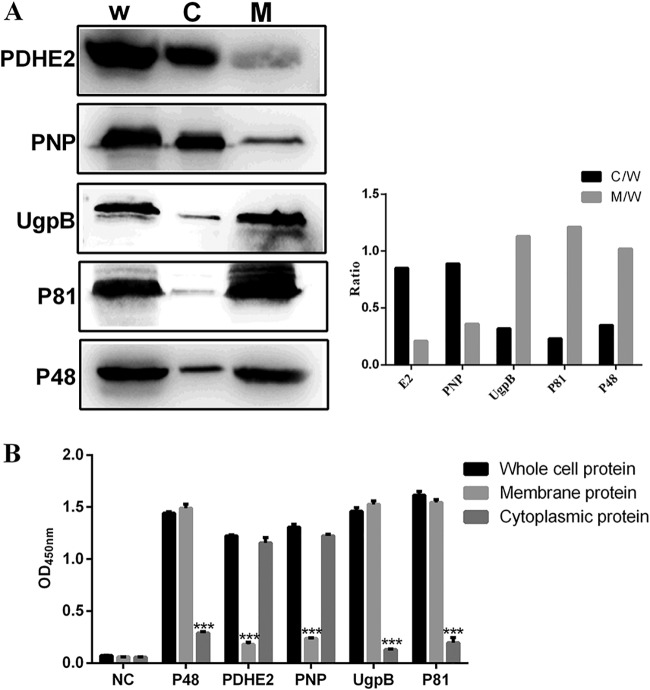
Subcellular localizations of PDHE2, PNP, UgpB, and P81 in M. bovis. (A) Western blotting with the listed specific antibodies. W, whole-cell protein; C, cytoplasmic protein; M, membrane protein. (Left) Representative blot; (right) graph showing the ratio of the protein amount in the cytoplasmic or membrane fractions to the total protein in whole-cell lysate. (B) For indirect ELISA, 96-well ELISA plates were coated with whole bacterial proteins, membrane proteins, or cytoplasmic proteins. Rabbit anti-PDHE2 serum, rabbit anti-PNP serum, rabbit anti-P81 serum, and rabbit anti-UgpB serum were used as primary antibodies, and HRP-conjugated goat anti-rabbit IgG was used as the secondary antibody. The optical density at 450 nm (OD_450_) was read. The whole-bacterial protein group was the control group. Data represent means ± SD from 3 independent experiments. Statistical analysis was done using a ratio-paired two-tailed *t* test and displayed only when significant. ***, *P* ≤ 0.001.

To identify the location of the four target proteins on M. bovis strain PD, Western blotting assays and iELISA were performed. In the Western blotting assay, PDHE2 and PNP were detected predominantly in the cytoplasm fraction and UgpB and P81 were detected predominantly in the membrane fraction (Fig. 3A). A monoclonal antibody (MAb) directed against known membrane protein P48 was included as a control (22). These findings indicate that UgpB and P81 are membrane-associated proteins, consistent with the iELISA results (Fig. 3B).

### MI-based identification of protective antigens *in vitro*.

To determine the protective antigens of M. bovis, we performed the MI test using rabbit antisera against the target proteins. The results indicate that rabbit anti-PDHE2 serum and anti-PNP serum had no inhibitory effect (Table 3). In contrast, rabbit anti-UgpB serum and rabbit anti-P81 serum each showed a high MI titer, whether rabbit serum (RS) or guinea pig serum (GPS) was used as the source of complement; 1:20 dilutions could inhibit the growth of 10^6^ CFU/ml of M. bovis strain PD (Table 3). Anti-M. bovis serum served as a positive control, and rabbit normal serum served as a negative control.

**TABLE 3:** MI titer and inhibitory dosage in MI tests^*a*^

Serum	Inhibitory dosage	MI antibody titer with indicated complement source	
		GPS	RS
Rabbit negative serum	<10	<8	<8
Rabbit anti-M. bovis serum	10^6^	256	512
Rabbit anti-PDHE2 serum	<10	<8	<8
Rabbit anti-PNP serum	<10	<8	<8
Rabbit anti-UgpB serum	10^6^	256	256
Rabbit anti-P81 serum	10^6^	1,024	1,024

a Inhibitory dosage, the concentration of the maximum dilution of M. bovis for which the medium remained red, was determined as the maximum mycoplasmacidal dosage with antiserum at a 1:20 dilution. MI titer is the concentration of the maximum antibody diluted at which the medium remained red.

### MI was detected by CFU determination.

**FIG 4:**
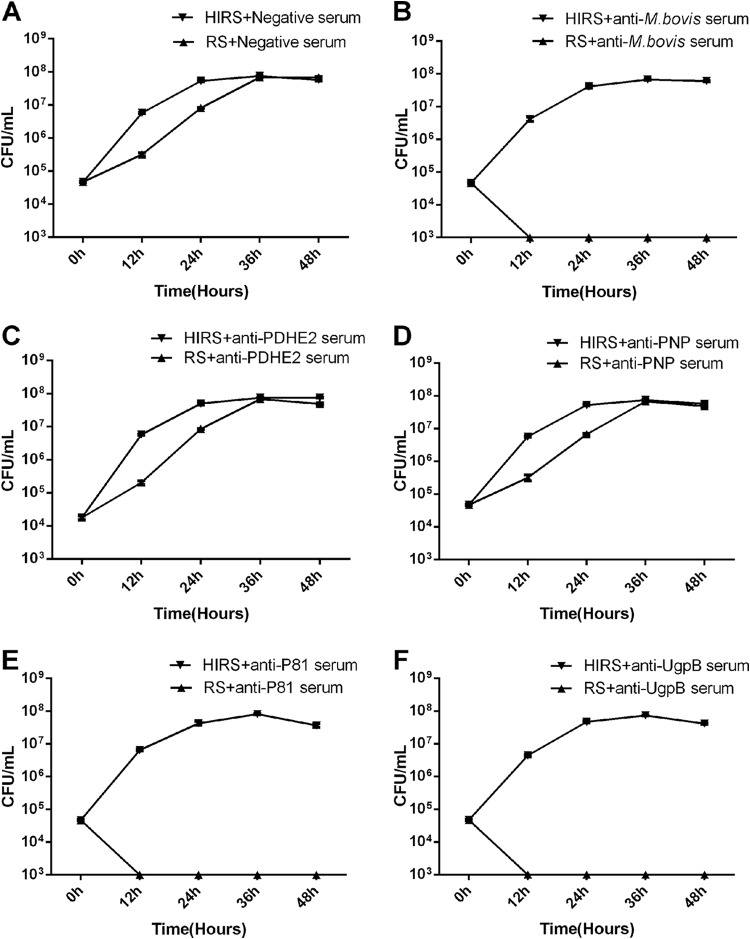
MI was detectd by CFU determination. All antisera were heated at 56°C for 30 min before use; fresh rabbit serum (RS) served as the source of complement, and heat-inactivated rabbit serum (HIRS) served as a control. Shown is growth of M. bovis in PPLO broth containing negative serum (A), anti-M. bovis serum (B), anti-PDHE2 serum (C), anti-PNP serum (D), anti-P81 serum (E), or anti-UgpB serum (F) at a dilution of 1:40 along with RS or HIRS (as the control group) at a dilution of 1:20. These data are presented as the means ± SD from three separate experiments.

Fresh normal RS served as a source of complement, and samples of M. bovis (∼5 × 10^4^ CFU/ml) were grown in complete PPLO broth medium containing one of various rabbit antisera. The color of the medium, which changes with M. bovis growth, was divided into categories corresponding to four growth stages (Table S2). As shown by the resulting M. bovis growth curves, the growth of M. bovis was only slightly inhibited in the presence of complement at 12 h after incubation, whether the medium contained negative serum (Fig. 4A) or antiserum against PDHE2 (Fig. 4C) and PNP (Fig. 4D). Notably, antibodies directed against the membrane proteins P81 (Fig. 4E) and UgpB (Fig. 4F) were as effective as antiserum against whole M. bovis cells (Fig. 4B) in the killing of M. bovis, which means nearly 100% killing (<10 CFU/ml) at 12 h after incubation in the presence of complement. In contrast, the effective killing in the presence of these antibodies was abolished when heat-inactivated rabbit serum (HIRS) was used instead of RS, suggesting that the metabolic inhibition of M. bovis is based on antibody-dependent complement-mediated killing, rather than on the inhibition of mycoplasma reproduction by antibodies separately.

### Complement killing.

**FIG 5:**
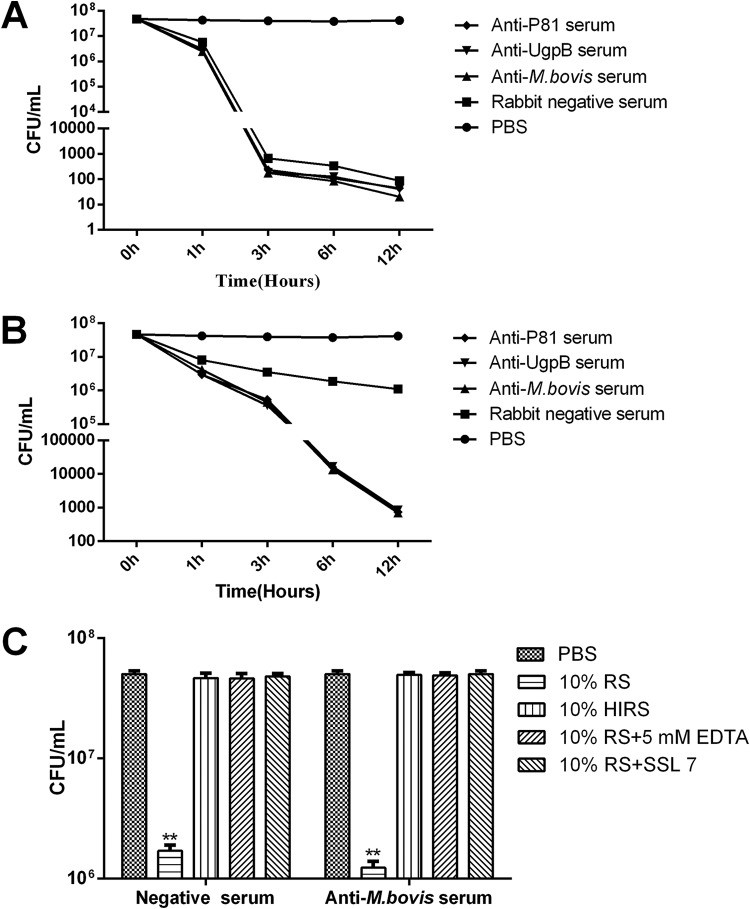
Complement killing assay. All antisera were heated at 56°C for 30 min before use, and fresh rabbit serum served as the source of complement. Complement killing assays were performed on M. bovis under the following conditions: antiserum was used at a 1:20 dilution and RS at a 1:10 dilution (A), antiserum was used at a 1:20 dilution and RS at a 1:40 dilution (B), or M. bovis was incubated with fresh rabbit serum in the presence of EDTA (5 mM) or SSL7 (40 μg/ml) (C). EDTA is the Mg^2+^ and Ca^2+^ chelator, which inhibits complement activation of all three pathways. The SSL7 protein from Staphylococcus aureus binds to C5 to inhibit complement-mediated hemolytic and bacterial activity. The PBS group was the control group. These data are presented as the means ± SD from three separate experiments. Statistical analysis was done using a ratio-paired two-tailed *t* test and displayed only when significant. **, *P* ≤ 0.01.

Complement killing assays were performed to further determine that mycoplasma growth could be inhibited by complement-mediated killing. In these experiments, fresh normal RS at a final dilution of 1:10 or 1:40 was used as the source of complement, and RS that had been heat inactivated was used as a control. Complement killing is a time-dependent process that requires several hours. A significant amount of M. bovis organisms were killed in the presence of hyperimmune rabbit anti-M. bovis serum and complement; the number of bacteria dropped from 5 × 10^7^ CFU at 0 h to 3 × 10^2^ CFU at 3 h. Notably, the differences in killing amount in the presence of complement among the anti-P81serum, anti-UgpB serum, and anti-M. bovis serum were not significant (Fig. 5A). Although fresh normal RS alone can kill M. bovis, the killing efficiency of complement is lower than the combined effect of antiserum and complement, especially at a low complement concentration (Fig. 5B). Heat treatment of serum at 56°C is a commonly used and straightforward method to inactivate certain heat-labile complement components. Next, addition of the chelating agent EDTA and specific inhibitor SSL7, to block the complement reaction, also interfered with complement killing, which suggests that the killing effect is complement mediated rather than merely due to antibody agglutination (Fig. 5C). Furthermore, these results also indicate that the antibody-dependent complement-mediated killing is more effective than that by the complement alone.

### Complement lysis.

**FIG 6:**
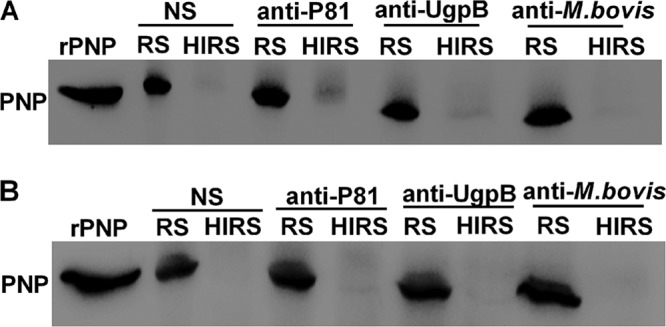
Complement lysis. All antisera were heated at 56°C for 30 min before use, and fresh RS served as the source of complement. Western blotting to detect the level of cytoplasmic protein PNP was performed to monitor the lysis of M. bovis following treatment with normal RS (NS), anti-P81 serum, anti-UgpB serum, or anti-M. bovis serum. M. bovis was incubated with RS at a 1:10 dilution as the source of complement or with HIRS for 1 h (A) or 3 h (B).

Complement ruptures the cell membrane, which leads to cytoplasm leakage of various cellular components, such as nucleic acid and cytoplasmic proteins. As a marker of complement lysis, PNP protein was detected by Western blotting. The results show that with RS as a source of complement at a final dilution of 1:10, there was some leakage of cytoplasmic proteins, after incubation at 37°C for 1 h (Fig. 6A) or 3 h (Fig. 6B), compared with findings for the heat-activated RS group.

### Membrane rupture of M. bovis strain PD.

**FIG 7:**
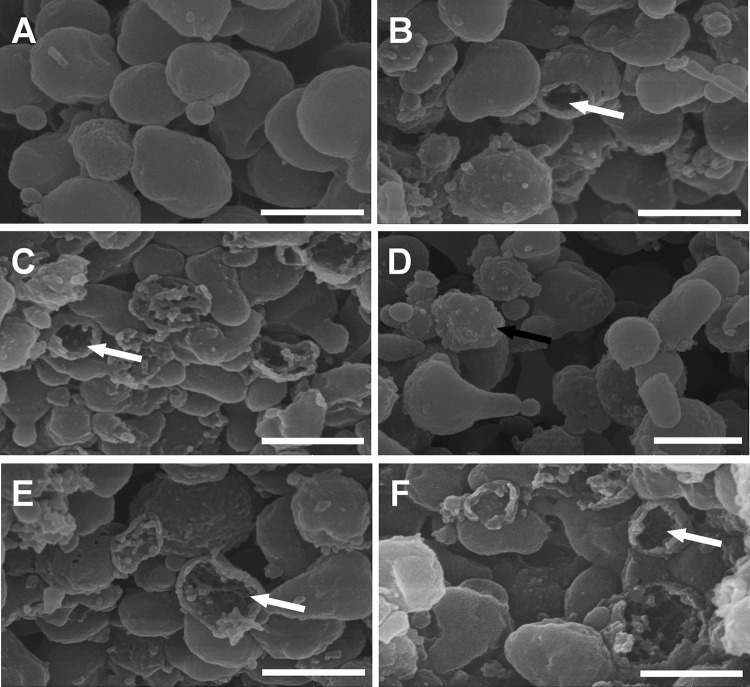
Scanning electron microscopy of M. bovis treated with various rabbit antisera. All antisera were heated at 56°C for 30 min before use, and fresh RS served as the source of complement. Shown are representative scanning electron microscopy images of M. bovis incubated with PBS (A), normal rabbit serum (B), rabbit anti-M. bovis serum (C), rabbit anti-M. bovis serum in the presence of heat-inactivated RS (D), rabbit anti-P81 serum (E), and rabbit anti-UgpB serum (F). The white arrows indicate large holes in ghost-like structures. The black arrow indicates a mycoplasma with a rough surface. Bar, 500 nm.

Massive M. bovis pellets were incubated with phosphate-buffered saline (PBS) and various heat-inactivated antisera at 37°C for 3 h in the presence of complement, and the results were analyzed by scanning electron microscopy. In the field of vision, M. bovis organisms suspended in PBS were polymorphic, with intact membranes (Fig. 7A). The addition of specific antibodies for M. bovis (Fig. 7C), membrane protein P81 (Fig. 7E), or UgpB (Fig. 7F) brought about a dramatic change in the morphology of the cells. We also observed a few ghosts (bacterial cells that had lost their cytoplasm) in M. bovis suspended in buffer with just untreated RS (Fig. 7B). Notably, the formation of ghosts was blocked by heat treatment of RS, although the mycoplasma surface was rough, in contrast to the smooth surface of control mycoplasmas (Fig. 7D).

## DISCUSSION

MI is commonly used for the measurement of growth-inhibiting antibody directed against acid-producing mycoplasmas, including M. bovis (23). Work by Eaton and colleagues has suggested that the metabolic inhibition of mycoplasmas by specific antiserum is analogous to virus neutralization; however, unlike for many viruses, the inhibition of mycoplasmas requires the presence of complement (24). In general, fresh GPS has been employed for this purpose. Additionally, mycoplasma medium containing non-heat-inactivated horse serum has been used in place of GPS as the source of complement (6). In this study, we found that the use of RS as a source of complement was similarly effective as the use of GPS, and RS is generally more accessible. Furthermore, our findings indicate that the metabolic inhibition of M. bovis is based on antibody-dependent complement-mediated killing.

To determine the major antigens involved in the immune killing of M. bovis, we used pulldown assays with rabbit anti-M. bovis antibodies as bait. Identification of the pulldown products by LC-MS/MS yielded nine different proteins, some of which are in line with the results obtained by two-dimensional (2D) gel electrophoresis and matrix-assisted laser desorption ionization–time of flight (MALDI-TOF) mass spectrometry in previous studies (25, 26). The identified antigens all had high immunogenicity and reactivity, qualities which could make them useful in the development of new diagnostic methods or vaccines for the treatment of M. bovis infection. Next, recombinant PDHE2, PNP, UgpB, and P81 were each expressed in an E. coli expression system, and the resulting proteins were used to prepare rabbit polyclonal antibodies. The MI results for these antisera suggest that the anti-P81 serum and anti-UgpB serum had high metabolism-inhibiting antibody titers, unlike the anti-PDHE2 serum and anti-PNP serum. Additionally, UgpB and P81 were found to be mainly distributed on the mycoplasma membrane surface, whereas PDHE2 and PNP were found to be mainly distributed in the cytoplasm. Together, the data from these studies suggest that mycoplasmacidal antibodies directed against certain M. bovis membrane proteins participate in the complement killing of these bacteria.

Complement is an important part of the innate immune system, contributing to the defense against invading bacteria through direct bacterial killing, leukocyte activation, or opsonization to attract phagocytic cells. Bacteria can activate all routes of complement activation; complement molecules recognize and bind to various structures on bacteria, resulting in the formation of C3 convertase enzymes on the bacterial surface (27). The MAC is formed, after which complement component C3b binds to the bacterial surface (28, 29). The abundant membrane proteins and carbohydrate chains on the surface of mycoplasmas may be potential sites of complement recognition. In this study, using complement killing and complement lysis assays, we observed that M. bovis organisms were killed directly by the complement system as a result of lysis of the organisms.

Previous work confirmed that antibodies can enhance phagocytosis through activation of the complement cascade and deposition of the complement component C3b on the pathogen surface (30). In this study, using serum bactericidal assays, we found that antibody-dependent complement-mediated killing is more effective than complement killing alone. This finding is also supported by a recent report on Bordetella pertussis and Neisseria meningitidis (31, 32). For most Gram-negative bacteria, lipopolysaccharide is classically associated with complement resistance, as the thick O-antigen structures prevent complement protein deposition onto the bacterial surface and hamper complement killing, but lipopolysaccharide is not sufficient to prevent antibody-dependent complement-mediated lysis (33). In experiments with Neisseria gonorrhoeae, the MAC was bactericidal only in the presence of specific bacterial antibody (34). One hypothesis posits that the antibody plays a stereospecific role in directing the attachment and deposition of complement components to specific sites on the bacterial surface, thus affecting the MAC insertion by which transmembrane channels are formed, but this idea has not yet been supported by experimental evidence. Studying the role of complement, and particularly of antibody-dependent complement-mediated killing of M. bovis, may be essential to understanding vaccine-induced protection and should be helpful for the design of improved vaccines.

Although the MAC can effectively kill a wide range of Gram-negative bacteria, including E. coli (17) and N. meningitidis (32), the exact mechanism of this process remains poorly understood. In recent years, erythrocyte membranes and artificial liposomes have been the most commonly used model systems for MAC lysis (35). Nevertheless, the MAC depends on the prior labeling of the bacterial surface with C5 convertase enzymes in bacterial killing, while it does not depend on C5 convertase enzymes in the lysis of liposomes or erythrocytes (36). Due to the characteristics of mycoplasmas, elucidating the exact mechanism of the lysis of M. bovis by the MAC is an effort worth investing in in the future.

In conclusion, our results show the following: M. bovis can be killed directly by the complement system as a result of lysis of the organisms, and the antibody-dependent complement-mediated killing is more effective than that by the complement alone. This study provides important data that could be useful for the prevention and treatment of M. bovis and the development of new vaccines. Lastly, we also demonstrated that mycoplasmas could be an appropriate new model for studying the lytic activity of the MAC.

## MATERIALS AND METHODS

### Bacterial strains and growth conditions.

M. bovis strain PD was isolated from the lungs of a bovine with pneumonia and subsequently cultivated in PPLO broth containing 2.1% (wt/vol) PPLO broth (Becton, Dickinson and Company, USA), 2.5% (wt/vol) yeast extract, 0.002% (wt/vol) phenol red, 20% heat-inactivated horse serum (HyClone, USA), and 1,000 U/ml of penicillin or on PPLO agar supplemented with 1% (wt/vol) agar at 37°C and 5% CO_2_.

Escherichia coli strains DH5α and BL21(DE3) (TransGen Biotech, China) were used for the expression of recombinant proteins and cultured in Luria-Bertani broth or on Luria-Bertani agar at 37°C. When necessary, ampicillin and kanamycin were added at 100 μg/ml and 50 μg/ml, respectively.

### Antibodies.

The following antibodies, all produced in-house, were used in our experiments: rabbit anti-M. bovis polyclonal antibody (pAb; dilution at 1:1,000), rabbit anti-PDHE2 pAb (dilution at 1:1,000), rabbit anti-PNP pAb (dilution at 1:1,000), rabbit anti-P81 pAb (dilution at 1:1,000), rabbit anti-UgpB pAb (dilution at 1:1,000), rabbit anti-P48 pAb (dilution at 1:1,000), and mouse anti-P48 MAb (dilution at 1:2,000).

The following commercially available antibodies were used as secondary antibodies in our experiments: horseradish peroxidase (HRP)-conjugated goat anti-mouse IgG (H+L) (ABclonal, China) and HRP-conjugated goat anti-rabbit IgG (H+L) (ABclonal, China) (both used at 1:5,000).

### Rabbit serum.

Rabbit serum (RS) was prepared from three healthy New Zealand rabbits. Pooled blood was collected in evacuated tubes, allowed to stand for 2 h at room temperature, and centrifuged for 10 min at 4,000 × *g* and 4°C. The complement in the serum was inactivated by incubating the serum in a water bath at 56°C for 30 min, or serum containing 40 μg/ml of SSL7 was used where indicated. The SSL7 protein from Staphylococcus aureus binds to C5 to inhibit complement-mediated hemolytic and bacterial activity (37, 38). The complement titers were calculated as the number of 50% hemolytic units of complement (CH_50_). All sera tested negative by iELISA using whole M. bovis organisms as the coating antigens. Serum was separated and stored at −80°C. A single lot of RS was used for all experiments in this study.

### Preparation of different components of bacterial proteins.

M. bovis was cultured in PPLO broth until the beginning of the stationary growth phase. The culture was then centrifuged for 20 min at 12,000 × *g*. The resulting pellet was washed three times, resuspended, and lysed in phosphate-buffered saline (PBS; 0.01 M, pH 7.4) by ultrasonic treatment for the extraction of all bacterial proteins (whole bacterial proteins).

Lipid-associated membrane proteins (LAMPs) were prepared by previously described methods (39). Briefly, bacterial pellets prepared as described above were resuspended in TBSE buffer (50 mM Tris, 0.15 M NaCl, 1 mM EDTA). Triton X-114 was added to a final concentration of 2%, after which the mixture was incubated for 60 min at 4°C. The lysate was then incubated for 10 min at 37°C for phase separation and centrifuged for 20 min at 12,000 × *g*. The upper aqueous phase was transferred to a new tube and replaced with an equal volume of TBSE. This phase separation was repeated twice. The final Triton X-114 phase was resuspended with TBSE to the original volume. To precipitate membrane lipoproteins, 2.5 volumes of ethanol were then added, and the sample was incubated overnight at 20°C. After centrifugation, the resulting pellet was resuspended in PBS and then split by a brief ultrasonic treatment.

The cytoplasmic proteins were prepared by using a nuclear and cytoplasmic protein extraction kit (Beyotime, China) in accordance with the product manual. In this method, the cells expand due to low osmotic pressure conditions, and the cell membrane eventually ruptures, releasing the cytoplasmic proteins.

### Pulldown assay.

To determine the major antigens involved in the immune killing of M. bovis, we performed a pulldown assay. First, protein A/G agarose was preincubated for 6 h at 4°C with 2 μg of rabbit-anti M. bovis antibodies or normal rabbit antibodies as a control and then washed three times with PBS (pH 7.4) via centrifugation for 5 min at 1,000 × *g* and 4°C. Second, the IgG-conjugated agarose was mixed with either the whole bacterial proteins or LAMPs and incubated for 12 h at 4°C. The mixture was then washed with PBS as described above. After centrifugation, the beads were resuspended with 30 μl of 1× SDS-PAGE loading buffer and boiled for 10 min before being subjected to electrophoresis on a 12% SDS-PAGE gel.

### LC-MS/MS identification of proteins.

The target protein bands were cut out after separation by SDS-PAGE and subjected to liquid chromatography-tandem mass spectrometry (LC-MS/MS). Briefly, each target protein band was dissolved in digest decolonizing solution (50% acetonitrile, 25 mM ammonium bicarbonate) and incubated with 10 mM dithiothreitol (DTT) for 1 h at 56°C. The mixture was then treated with 55 mM iodoacetamide (IAM) for 45 min at room temperature and washed twice with 25 mM ammonium bicarbonate. After acetonitrile dehydration and trypsin digestion, the sample was desalted with a Prominence nano 2D system (Shimadzu, Japan) equipped with a C_18_ reverse-phase column (Eprogen, USA). The peptides were eluted by a gradient mode with 5 to 80% acetonitrile in 0.1% formic acid over 60 min at 400 nl/min. Mass spectra were recorded by a MicrOTOF-QII (Bruker Daltonics, USA). Data were acquired in a data-dependent mode using the Bruker Daltonics micrOTOF control system. The strongest peaks of each MS acquisition were selected for use in the following MASCOT search. The MS/MS spectra were processed by Data Analysis 3.4 (Bruker Daltonics, Germany) with a signal/noise ratio (S/N) of ≥4.0 and automatically searched against the IPI.RAT database (version 3.41) using Mascot 2.1.0 (Matrix Science, UK). The NCBI database was also used in this study.

### Expression and purification of the recombinant proteins.

The target genes were amplified from M. bovis strain PD chromosomal DNA. The specific primers for gene point mutations used here in this study are listed in Table S1. The resulting PCR products were digested with the restriction enzymes BamHI and XhoI (New England BioLabs Inc., USA) and then cloned into the pET28a(+) vector using T4 DNA ligase. The resulting recombinant plasmids were separately transformed into Escherichia coli BL21(DE3). Expression of the target proteins was induced by the addition of 1 mM isopropyl-β-d-thiogalactoside (IPTG) for 6 h at 37°C. The recombinant proteins were purified with HisPur nickel-nitrilotriacetic acid (Ni-NTA) resin (Thermo Scientific, USA) and then analyzed via SDS-PAGE and Western blotting with rabbit anti-M. bovis IgG or anti-M. bovis Ab-positive bovine serum. The concentration of each protein was determined with a bicinchoninic acid (BCA) protein assay kit (Cwbiotech, China) before the protein was stored at −20°C for later use.

### Preparation and determination of rabbit polyclonal antibody.

Each purified recombinant protein was separately injected subcutaneously into New Zealand rabbits (*n* = 3) at a dose of 1 mg/kg of body weight in Freund’s complete adjuvant. Two additional inoculations with 2 mg/kg of protein in incomplete Freund’s adjuvant were performed after 2 and 4 weeks. Serum was harvested 10 days after the last injection, and the titer of the serum was detected via indirect enzyme-linked immunosorbent assay (iELISA) using M. bovis extracts as the coating antigens as well as by agar gel diffusion precipitation and indirect hemagglutination assay, according to a standard molecular biology technique (25). Ammonium sulfate precipitation and dialysis desalination were used to extract pAbs. The concentration of the resulting antibody was determined with the BCA protein assay kit (Cwbiotech, China) before the antibody was stored at −20°C for future use.

### iELISA.

Indirect ELISA was performed as described previously (26). Briefly, 96-well ELISA plates were coated with 100 ng/well of whole bacterial proteins, membrane proteins, or cytoplasmic proteins and allowed to incubate at 4°C overnight. After being washed three times with PBST (0.01 M PBS with 0.05% [vol/vol] Tween 20), the plates were blocked with 5% skim milk in PBST for 2 h at 37°C. Sera (primary antibodies) were diluted as required, and 100 μl/well was incubated at 37°C for 1h. After a washing, HRP-conjugated goat anti-rabbit IgG secondary antibody was diluted to 1:5,000 (vol/vol). Finally, the substrate tetramethylbenzidine (TMB) and 2 M H_2_SO_4_ were added for coloration and termination of the reaction, respectively. The plates were read at an optical density of 450 nm (OD_450_) with an ELISA plate reader (Pharmacia, USA).

### MI test.

The metabolism inhibition (MI) test to determine growth-inhibiting antibody titer was performed in 96-well disposable plastic microtiter plates as previously described (40, 41). Briefly, antiserum was diluted 1:4 in complete PPLO broth and heated at 56°C for 30 min before use. After the addition of 25 μl of complete PPLO broth with additives to each well, the serum was serially diluted 2-fold. M. bovis suspensions (50 μl, diluted as required) were added into each well, and the total volume was brought up to 200 μl by the addition of 125 μl of PPLO broth containing 10% guinea pig serum (GPS) or RS. After 48 h of incubation at 37°C, the color of the culture medium was checked. The concentration of the maximum antibody diluted at which the medium remained red was determined as the MI titer. This assay was repeated three times.

All antiserum was heated at 56°C for 30 min and then diluted 1:20 in complete PPLO broth along with 100 μl of RS as a source of complement. M. bovis suspensions were serially diluted 10-fold, and 1 ml of each dilution was added per tube. The total volume was brought up to 2 ml. After 48 h of incubation at 37°C, the color of the culture medium was recorded. The concentration of the maximum dilution of M. bovis for which the medium remained red was determined as the maximum mycoplasmacidal dosage. This assay was repeated three times.

### CFU determination.

The metabolism inhibition assay was performed in test tubes, the color of the medium was observed, and the number of CFU was determined after incubation for various periods at 37°C. Fresh RS served as a source of complement in the experimental group. The reaction mixture consisted of 1 ml of M. bovis suspension (∼2 × 10^4^ CFU/ml), 50 μl of heat-inactivated antiserum, 100 μl of RS, and enough complete PPLO broth to bring the total volume up to 2 ml. The mixture was incubated at 37°C, after which the color of the culture medium was recorded and the number of CFU was determined. Heat-inactivated normal RS was employed as the negative-control group.

### Complement killing.

Complement killing of M. bovis was performed as previously described (29). Briefly, M. bovis (about 5 × 10^7^ CFU) suspended in reaction buffer (PBS containing 5 mM Mg^2+^ and 0.5 mM Ca^2+^) was incubated with heat-inactivated antiserum at 37°C for 30 min, to which different dilutions of RS were added as the source of complement. Samples were withdrawn immediately or at various time intervals after the addition of complement. Heat treatment of serum at 56°C was used to inactivate certain heat-labile complement components, or specific inhibitors SSL7 and EDTA were used to block the complement reaction. To stop the reaction, the samples were diluted 1:10 in PBS (0.01 M, pH 7.4) and put on ice. The samples were then serially diluted 10-fold in PBS and plated onto PPLO agar plates at 37°C and 5% CO_2_ for the enumeration of CFU after 3 days of incubation. Three samples were assayed for each strain in each experiment.

### Complement lysis.

Fresh intact M. bovis strain PD cells were harvested at mid-log phase and washed three times with PBS (0.01 M, pH 7.4). Reaction mixtures were prepared as described above for the complement killing test. After incubation at 37°C for 1 h or 3 h, the samples were centrifuged at 13,000 × *g* for 5 min, and the resulting supernatants were transferred to tubes for used in a Western blot analysis using rabbit anti-PNP antibody.

### Western blotting.

After SDS-PAGE electrophoresis on 12% gels, the target protein bands were transferred onto polyvinylidene difluoride (PVDF) membranes (Millipore, USA) for 90 min at 120 V. After being washed three times with PBST (0.01 M PBS with 0.05% [vol/vol] Tween 20), the membrane was blocked with 5% skim milk in PBST for 2 h at 37°C, incubated with primary antibodies (diluted in PBST) for 60 min at 25°C, and washed three times with PBST, followed by incubation with secondary antibodies (diluted in PBST). After being washed three times, the blots were visualized with ECL reagents (High-sig ECL substrate; Tanon, China), and images were collected with a Tanon-5200 chemiluminescent imaging system (Tanon, China). For the quantification of protein levels, the density of bands was determined with ImageJ.

### Scanning electron microscopy.

Fresh intact M. bovis strain PD cells were harvested at mid-log phase, washed three times with PBS (0.01 M, pH 7.4), and incubated with heat-inactivated antiserum diluted 1:20 in PBS for 3 h at 37°C, with addition of fresh RS as the source of complement. After the incubation, the samples were centrifuged for 20 min at 12,000 × *g*. The resulting pellet was prefixed overnight in 2.5% glutaraldehyde in PBS, washed with PBS three times, and then sequentially dehydrated with 30%, 50%, 70%, 80%, 90%, and 100% ethanol in distilled water for 15 min each. After dehydration, the samples were dried using a critical point drying system and coated with a 2-nm platinum palladium film in a sputter coater. The samples were then observed using a scanning electron microscope, model SU8010 (Hitachi, Japan), at an accelerating voltage of 10 kV.

### Ethics statement.

All animal studies were performed in accordance with the China Agricultural University Institutional Animal Care and Use Committee guidelines (CAU20180401-2) and followed the International Guiding Principles for Biomedical Research Involving Animals. Experiments were approved by the Beijing Administration Committee of Laboratory Animals.

### Statistical analysis.

GraphPad Prism 6.01 (GraphPad Software) was used for graph design and statistical analyses. Statistical analysis was done using a ratio-paired two-tailed *t* test as indicated in the figure legends. All experiments were performed in triplicate, and data are given as means ± standard deviations (SD).

## Supplementary Material

Supplemental file 1
