# Supplementary material for: Antibodies Specific to Membrane Proteins Are Effective in Complement-Mediated Killing of Mycoplasma bovis
Source: Infect Immun. 2019 Nov 18;87(12):e00740-19. doi: 10.1128/IAI.00740-19 (PMC6867846; doi:10.1128/IAI.00740-19)
Supplement: Supplemental file 1 [file IAI.00740-19-s0001.pdf]

Supplementary Material

Table S1. Primers for PCR analysis

Number	Primer sequence	Position
Pyruvate dehydrogenase E2 component(PDHE2)		
E2-F	GGATCCATGTTTAAAATGCAATTTACAGACG	1→25
E2-R	CTCGAGTTAGTTTGTTTCAATGTGAAAAATTT	212←237
Purine-nucleoside phosphorylase(PNP)		
PNP-F	GGATCCATGACACCACACATAAATGCACC	1→23
PNP-R	CTCGAGTTATGCTAACTCTAAAGCTATTTTC	677←702
glycerol ABC transporter(UgpB)		
ugpB-1F	GGATCCATGTTGAAAAAAATTTTTATTAAAGCTG	1→28
ugpB-1R	GCTTTTAAAGCATA**CCA**AATTGATTGTTC	679←707
ugpB-2F	GAACAATCAATT**TGG**TATGCTTTAAAAGC	679→707
ugpB-2R	GAATTATTGTAGTCTTT**CCA**TAATTCTTGG	999←1028
ugpB-3F	CCAAGAATTA**TGG**AAAGACTACAATAATTC	999→1028
ugpB-3R	AGATTTG**CCA**TGAACC**CCA**TTCTTTTG	1115←1141
ugpB-4F	CAAAAGAA**TGG**GGTTCA**TGG**CAAATCT	1115→1141
ugpB-4R	TGTA**CCA**GTTATCTTCTTCTTCGCCAGAAACCTTGTT TTTGCCTGTGTATAT**CCA**TTTA	1416←1474
ugpB-5F	TAAA**TGG**ATATACACAGGCAAAAACAAGGTTTCTG GCGAAGAAGAAGATAAC**TGG**TACA	1416→1474
ugpB-5R	CTCGAGTTATTGTTTTTTAGTTTGTTCAAATG	1853←1857
membrane lipoprotein P81		
P81-1F	GGATCC ATGAGTAAGAAAAATAAATTAATGATTG	1→28
P81-1R	AATGAAGCCATAGTATT**CCA**TTGTGGCT	155←182
P81-2F	AGCCACAA**TGG**AATACTATGGCTTCATT	155→182
P81-2R	TTTCAAATTCACC**CCA**TAATTTTTGTACT	675←703
P81-3F	AGTACAAAAATTA**TGG**GGTGAATTTGAAA	675→703
P81-3R	TCTTAAGAATAATAG**CCA**TTGTTTATTATC	1441←1470
P81-4F	GATAATAAACAA**TGG**CTATTATTCTTAAGA	1441→1470
P81-4R	TCTTTTCATTAGATTTATT**CCA**TTTACCAG	1664←1693
P81-5F	CTGGTAAA**TGG**AATAAATCTAATGAAAAGA	1664→1693
P81-5R	CTCGAG TTATTTAAGAATTTGACTGCTTGATG	2162←2187

Underline indicates introduced restriction enzyme sites. Bold indicates TGA-to-TGG changes of the codon for permitting tryptophan expression in *E. coli*.

Table S2. Association between color change and mycoplasma growth time.

Fresh normal RS severed as a source of complement, and samples of *M. bovis* were grown in complete PPLO broth medium containing one of various rabbit antisrum. The color of the medium, which changes with *M. bovis* growth, was divided into categories corresponding to four growth stages.

	NC	PC	NS		anti-*M.bovis*		anti-PDHE2		anti-PNP		anti-P81		anti-UgpB	
			RS	HI	RS	HI	RS	HI	RS	HI	RS	HI	RS	HI
0h	-	-	-	-	-	-	-	-	-	-	-	-	-	-
12h	-	+	-	+	-	+	-	+	-	+	-	+	-	+
24h	-	++	+	++	-	++	+	++	+	++	-	++	-	++
36h	-	+++	++	+++	-	+++	++	+++	++	+++	-	+++	-	+++
48h	-	+++	+++	+++	-	+++	+++	+++	+++	+++	-	+++	-	+++

NC:negative control,2mL PPLO broth; PC: positive control, 2mL PPLO broth containing 5×10^4 CFU/mL *M.bovis*. RS: with normal rabbit serum as the complement source; HI: with heat-inactivated normal rabbit serum as control. Grade of color: -, red; +,yellowish red; ++, orange; +++, yellow.

Figure Captions

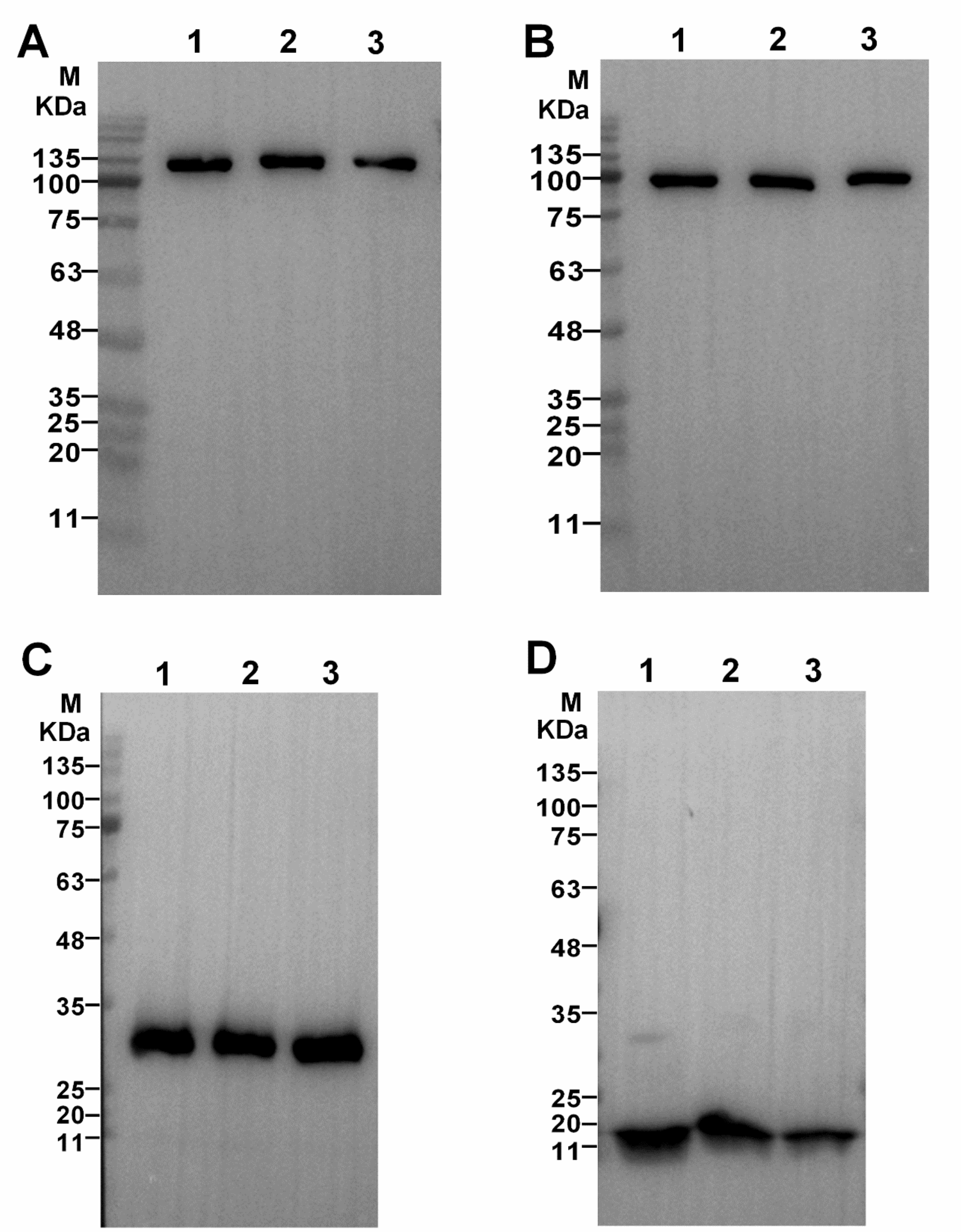

Fig.S1. Western blotting analysis. Western blotting was conducted with 25 μg of whole cell proteins from *M.bovis* PG45 (Lanes 1), PD (Lanes 2), HB (Lanes 3), using rabbit anti-P81 pAb (A), rabbit anti-UgpB pAb (B), rabbit anti-PNP pAb (C), rabbit anti-PDHE2 pAb (D) as the primary antibody, respectively.
